# On Treatment of Scarlatina by Inunction

**Published:** 1851-03

**Authors:** 


### ARTICLE V.

*On the treatment of Scarlatina by Inunction*, by Dr. SCHNEEMAN. (*London Journal of Medicine*, Sept. 1850.)

[In Vol IX, p. 15, of our "Abstract," we briefly mentioned this treatment on the authority of Mauthner, a physician of high repute in Vienna. We here present our readers with a more detailed accounnt; premising, that however puerile this treatment may appear, *prima facia*, its harmlessness may entitle it to a trial where a more hazardous proposition might reasonably be rejected. The following account is abridged from the "Lancet," Sept. 15, 1849.]

*Description of the Treatment.*—As soon as we are certain as to the nature of the illness, the patient must be rubbed every morning and evening, over the whole body, with a piece of bacon, in such a manner that, with the exception of the face (?) and hairy scalp, a covering of fat is everywhere applied. In order to make this rubbing-in somewhat easier, it is best to take a piece of the size of the hand, choosing a part still armed with the rind, that we may have a firmer grasp. On the soft side of this piece, slits are to be made in various directions, in order to allow the oozing out of the fat; and this is still further promoted by placing the bacon, for some time previously to using it, near the stove, in the oven, or on the hob. But the fat must be allowed to cool before being used.

The rubbing must be so performed, that the skin may be regularly, but not too quickly saturated with fat. During the process, only that part being rubbed is to be uncovered, or the whole can be done under the bedclothes; but this precaution is unnecessary. Although this plan, from the mess it makes, is not calculated to find favor, as it dirties bed and linen, as well as the persons of the children, yet the first few days yield results which make all this forgotten, and inspire the mothers with enthusiasm. With rapidity the most painful symptoms of the disease are allayed; quiet, sleep, appetite, and good humor return. Other things, however, are necessary besides infriction with fat; but still the most important share of the merit may be imputed to this treatment. The truth of this will appear from a citation of the first results which follow :

1. The improbability, I might say impossibility, of the patient getting cold. Were this the sole benefit of the inunction, it would be great.

2. The dry brittleness of the skin, and the tormenting itching, are not only materially alleviated, but, for the most part, fully put a stop to. Hence children generally like the rubbing-in, and often ask for a repetition of it before the time is come.

3. The influence on the physiological functions of the skin is still more important. During the coming on of scarlet fever, the skin becomes diseased, in consequence of which it dies off; and, until a new covering is prepared for the surface, the functions of the skin are ill performed, or, during the desquamation, probably not performed at all. To appreciate the importance of the imperceptible functions of the skin, merely mechanically viewing the matter, I refer to the experiments of Seguin, which fix the quantity of matter thrown off from the outer skin at eleven grains per minute in a grown person, and therefore more than two pounds per day. What effort it must cost the organism to lead so large a quantity into other paths, in order to throw it off when the skin is incapable of doing so! To give a striking proof of the bad influence which the interrupted functions of the skin produce on the healthy activity of relative, even of distant organs, I may cite the fact, that death is always the result where more than one half of the skin has been destroyed by fire or boiling liquid. A similar destruction of the skin ensues in scarlet fever, with this difference, that it takes place gradually, and thereby the organism is better enabled, by employing all the activity of the body, to find aid against the mischief which, to the injury of the patient, must result from the cessation of the functions of the skin.

4. The oxidation of the blood is thus considerably promoted, the interrupting of which is the cause of such serious phenomena. As the disease of the throat is not improbably, in great part, due to the interruption of the functions of the skin and lungs, it must naturally disappear, or not present itself, where these are continued in integrity; and such is found, in practice, to be the case.

5. Owing to the fatty covering, the skin is kept moist, and the cuticle cannot be driven about the room by currents of air; and thus one fertile source of infection is kept closed up, it being well known that infection is most easily communicated at the period of desquamation. The danger of infection, under any circumstances is materially lessened with the disappearance of the eruption from the skin, inasmuch as the process of generating infectious material is interrupted by restoring the skin to its normal state.

6. By shortening the period of desquamation, and protecting against the sequela of the disease, the duration of the treatment can be shortened to a period of from six to ten days.

7. The remedy is (cheap,) harmless, practical, and is perhaps never counter-indicated. The linen must not be too frequently changed, as a clean shirt takes up more of the fatty matter than one already saturated, and hence the skin is sooner deprived of its fatty covering. The rubbing-in is to be kept up twice a day for three weeks, and once a day during the fourth. The patient is, after this, to be washed daily with cool water and soap, and then only is the warm bath to be commenced. This process does not interfere with the natural course of the malady, and expose the individual to second and third attacks. In severe cases, the remedy may be repeated three or four times within the twenty-four hours. The main point is, to keep the skin *always* cool and moist; and here, even with all possible precaution, the skin will sometimes come off in certain places. The practitioner will do well to fix the exact hours at which the rubbing-in is to take place: it will then most probably be better and more regularly performed.

Other points, which are also important enough, now remain to be noticed.

8. *Temperature.*—Experience proves that it requires no great daring to keep the patient cold instead of hot. Cold washing is *not* to be employed, as it promotes desquamation. Cool air seems to have a bracing influence on the system, and a soothing one on the respiration; and all danger is avoided by the fatty covering. The temperature of the bedroom should never be above 13°R. The idea of throwing back the eruption by mere cold air is an error. Great heat is much more likely than cold to throw in the eruption. In fact, the fatal cases of this kind are principally those where, through keeping the patient too hot, the cerebral affection has been brought on; this has given rise to paralysis which appears sooner in the skin than in other parts, and thus to the withdrawal of the eruption—for the skin dies sooner than other parts, as shown in collapse, where mustard poultices do not act as in other states.

9. *Bed.*—The patient should not remain in bed any longer than is absolutely necessary. As soon as the fever, headache, and a desire to remain in bed are gone, he may quit it, for in bed the skin is between two fires,* and the functions of the body do not go on so well as in moving about. Hence, even when the patient must lie down an hour or two daily, he should still go about the rest of the time.

10. *Diet.*—The diet should be light, but there must be no starvation, and as rapid a return as possible to the usual food.

11. *Washing.*—Although it brings on desquamation, it will be as well to let the patient occasionally wash his hands and face with water and soap. It reconciles him to the dirt attendant on the rubbing-in.

12.—If *constipation* ensue, it must only be combated with medicines; when, at the end of forty-eight hours, it makes no semblance of disappearance, then a clyster of poppy oil is the best remedy.

The author enters his protest against a partial employment of his remedies.

*Complications require a modification of the system.*—1. *Severe cerebral symptoms at the commencement.*—The above treatment can only be applied where time is allowed for the development of the restorative process. Occasionally the case is accompanied at its very outset by severe cerebral affection, and convulsions. Here bleeding may be employed, and if necessary, unhesitatingly repeated. To support this view, Dr. Schneemann instances the authority of Armstrong, Bernd, Stieglitz, Hammond, Hingeston, &c. Venesection is, accordingly, the sheet anchor. The other remedies are:—The application of concentrated cold to the head; and the best form for this is ice. The cold dash is often more hurtful than useful, on account of the serious reaction which follows it, and exposes the patient more to the dangers of an apoplectic attack, although its results for the minute are often very flattering. *At the end of every two hours the bladder of ice should be removed*, in order that the uninterrupted effect of the cold may not weaken too much the tone of the vessels of the brain. Warm mustard plasters to the shins are a most valuable remedy. Internal remedies are generally of little use where the above given remedies fail; the only one of any importance is the carbonate of ammonia. Mercury is of little value, except

* Here it will be as well to state that in Germany a feather bed is frequently substituted for bed-clothes.

just to open the bowels; for its specific action never comes into full play till the system is throwing off the affection. There are, however, much better purgatives than calomel; the saline, for instance. Emetics ought not to be tried in cases complicated with cerebral affection; in others they may. The aconite failed in the author's hands, both in tincture and solution. With regard to the treatment by leeches and ammonia, so many writers have already pointed out its good results, that the author can safely recommend it, but with the proviso that in urgent cases bleeding be substituted for leeches.

2. *The Affection of the Throat.—Primary.* As this is but a link in the whole, so must the measures taken for its removal be such as will remedy the general affection. *Secondary.* For this, the rubbing-in, as it acts by prophylaxis, is the best possible remedy; but where this has not been brought into use, or where, from keeping the patient too warm, desquamation has come on, and the secondary sore throat has set in, the best remedy is the use of emetics. They not only remove the tough glazy slime, but excite the secretions and excretions to more normal disposition, and this is especially the case if the disorder have a gastric character. Many, by confounding the employment of emetics in the early and latter stages, have brought them into discredit. For the swelling of the tonsils, an excellent remedy is a solution of nitrate of silver (twenty grains to an ounce of water), with which they and the soft palate are to be painted; but so many varieties present themselves in these secondary attacks following on scarlet fever, that no general rules can be laid down. Here everything depends on the discrimination and judgment of the practitioner.

*Prophylaxis.*—Warm clothing, separation from school, and above all, light diet, are the best preventatives. That the younger children, as more predisposed than the others, must be more carefully separated from the sphere of infection, is doubtful practice. Not only are many children never infected, who, however, by adopting this system, require to be as carefully secluded as the others, thus causing great inconvenience; but the mildening influence and actual advantage of a gradual accustoming to the infection are thereby lost, and consequently the attack when it comes, is so much the more severe. The free communication with the patient is good, in order that constant exposure may, as in the case of physicians and nurses, blunt and wear out the disposition to the disease; for as it now is, children are separated from the patient during the first period, which is the mildest, and exposed to contact with him during the process of desquamation, which is the source of the most dangerous infection. Many preservatives have been vaunted against this malady during its epidemic appearance; of these, however, the only one apparently deserving of much credit is the belladonna. Its action appears to be, "by altering the relation of the nervous system, to diminish the disposition of the disease." The author's prescription is—Take of extract of belladonna one to two grains; distilled water, one ounce. Mix. Give to each child, morning and evening, as many drops as it has years, and continue to do so at least fourteen days. But not to confine himself to his own testimony, Dr. Schneemann gives that of Jordens, Ettmuller, Hedenus, Gumpert, Hufeland, Martius, Pormey, Behr, Benedix, Thaer, &c., who have testified to the value of belladonna; and though he admits that as many opponents might be found, he thinks it scarcely credible that all the former had deceived themselves. In certain sections of the circle of Bayeux, Dr. Feron preserved from scarlet fever all the children who had not been attacked before he commenced operations. In 400 cases near Valenciennes, treated with belladonna, not one person was attacked.—*Ranking's Abstract.*

